# The comprehensive ‘Communicate to Vaccinate’ taxonomy of communication interventions for childhood vaccination in routine and campaign contexts

**DOI:** 10.1186/s12889-017-4320-x

**Published:** 2017-05-10

**Authors:** Jessica Kaufman, Heather Ames, Xavier Bosch-Capblanch, Yuri Cartier, Julie Cliff, Claire Glenton, Simon Lewin, Artur Manuel Muloliwa, Afiong Oku, Angela Oyo-Ita, Gabriel Rada, Sophie Hill

**Affiliations:** 1 School of Psychology and Public Health, La Trobe University, Melbourne, Australia; 2 Norwegian Institute of Public Health, Oslo, Norway; 3 Swiss Tropical and Public Health Institute, Basel, Switzerland; 4 University of Basel, Basel, Switzerland; 5 International Union for Health Promotion and Education, Saint-Maurice Cedex, France; 6 Eduardo Mondlane University, Maputo, Mozambique; 7 Norwegian Institute of Public Health, Oslo, Norway; 8 South African Medical Research Council, Cape Town, South Africa; 9 Provincial Directorate of Health, Nampula, Mozambique; 10 University of Calabar, Calabar, Nigeria; 11 Pontifical Catholic University of Chile, Santiago, Chile

**Keywords:** Communication, Childhood vaccination, Immunisation, Taxonomy, Interventions, Campaigns

## Abstract

**Background:**

Communication can be used to generate demand for vaccination or address vaccine hesitancy, and is crucial to successful childhood vaccination programmes. Research efforts have primarily focused on communication for routine vaccination. However, vaccination campaigns, particularly in low- or middle-income countries (LMICs), also use communication in diverse ways.

Without a comprehensive framework integrating communication interventions from routine and campaign contexts, it is not possible to conceptualise the full range of possible vaccination communication interventions. Therefore, vaccine programme managers may be unaware of potential communication options and researchers may not focus on building evidence for interventions used in practice.

In this paper, we broaden the scope of our existing taxonomy of communication interventions for routine vaccination to include communication used in campaigns, and integrate these into a comprehensive taxonomy of vaccination communication interventions.

**Methods:**

Building on our taxonomy of communication for routine vaccination, we identified communication interventions used in vaccination campaigns through a targeted literature search; observation of vaccination activities in Cameroon, Mozambique and Nigeria; and stakeholder consultations. We added these interventions to descriptions of routine vaccination communication and categorised the interventions according to their intended purposes, building from an earlier taxonomy of communication related to routine vaccination.

**Results:**

The comprehensive taxonomy groups communication used in campaigns and routine childhood vaccination into seven purpose categories: ‘Inform or Educate’; ‘Remind or Recall’; ‘Enhance Community Ownership’; ‘Teach Skills’; ‘Provide Support’; ‘Facilitate Decision Making’ and ‘Enable Communication’. Consultations with LMIC stakeholders and experts informed the taxonomy’s definitions and structure and established its potential uses.

**Conclusions:**

This taxonomy provides a standardised way to think and speak about vaccination communication. It is categorised by purpose to help conceptualise communication interventions as potential solutions to address needs or problems.

It can be utilised by programme planners, implementers, researchers and funders to see the range of communication interventions used in practice, facilitate evidence synthesis and identify evidence gaps.

**Electronic supplementary material:**

The online version of this article (doi:10.1186/s12889-017-4320-x) contains supplementary material, which is available to authorized users.

## Background

Communication features in most vaccination programmes and activities. Vaccination communication may be used to generate demand for routine vaccination, facilitate the introduction of new vaccines, or publicise vaccination campaigns [1–6]. It can change how people think and feel about vaccination and is instrumental in addressing vaccine hesitancy [7–9]. However, communication is not always considered, planned or delivered in a rigorous and evidence-informed way [7, 10].

One potential reason for this is that communication is not often seen as a health intervention in its own right [11]. In fact, there is a broad range of potential vaccination communication strategies that can have meaningful impacts on individual and population health and behaviours. Until recently, there has been no coherent framework available for conceptualising these interventions. This lack of a conceptual overview for vaccination communication means that programme managers may not be aware of all the interventions that might be available to them, and researchers and research funders cannot focus their energy on building evidence for communications strategies that programmed managers are using and innovating in the field.

**Fig. 1:**
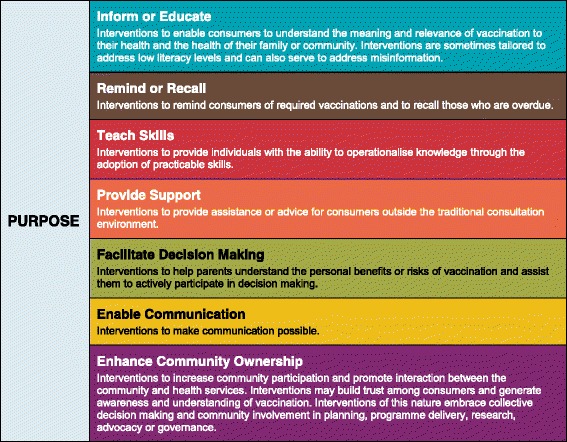
The COMMVAC routine vaccination communication taxonomy purposes

In our initial effort to address this problem and illuminate the diverse vaccination communication interventions, the ‘Communicate to Vaccinate’ (COMMVAC) project [12] developed a taxonomy or classification system of communication interventions related to routine childhood vaccination (‘the routine vaccination taxonomy’) [5]. This taxonomy was developed in a systematic way, drawing interventions from a variety of data sources including high-quality trial research as well as the experiences and perspectives of international vaccine experts and practitioners [5]. We defined ‘routine vaccination’ as the delivery of childhood vaccines recommended by the WHO [13]. The routine vaccination taxonomy focused primarily on communication that involved or impacted consumers (parents, caregivers or community members). It organised the interventions according to seven intended purposes (Fig. 1) and for three target groups (parents, community members, health providers), giving vaccination stakeholders a consistent way to describe, distinguish and conceptualise routine vaccination communication. In Fig. 1, we present an overview of the purposes from the routine vaccination taxonomy.

In addition to providing programme planners and researchers with an overview of existing interventions, the COMMVAC team used the routine vaccination taxonomy to map existing research evidence and inform consultations with international stakeholders to prioritise topics for two systematic reviews [5, 14, 15].

However, like most research related to vaccination communication, the COMMVAC taxonomy focused on routine vaccination. Routine childhood vaccination is the goal for sustainable vaccination programmes, but the reality is that many low- and middle-income countries (LMICs) still rely on large-scale vaccination campaigns such as supplementary immunisation activities (SIAs) to achieve and maintain coverage rates, address outbreaks or work towards disease eradication [16]. A vaccination campaign is an organised effort to deliver a vaccine or vaccines to a large number of people at one or more locations in a short time [17]. Campaigns tend to be well-resourced and utilise communication in a variety of forms [18, 19]. To our knowledge, there have been no attempts to develop a framework for organising the range of communication interventions in campaign activities.

Therefore, building on our routine vaccination taxonomy, we systematically identified the communication interventions used in campaigns for childhood vaccines and developed a comprehensive COMMVAC taxonomy of childhood vaccination communication interventions for any vaccination context.

### Aim and objectives

This paper aims to present a comprehensive taxonomy of communication interventions for childhood vaccination that broadens the scope of the routine taxonomy to include communication used in vaccination campaigns. Our objectives were:

1. To identify communication interventions used in vaccination campaigns through literature searches, observation in LMIC settings and consultation with vaccination stakeholders;
2. To revise the routine vaccination taxonomy categories, definitions and structure to include campaign communication interventions and incorporate stakeholder feedback.

## Methods

The methods used to develop the routine vaccination taxonomy have been described elsewhere [5]. To create the comprehensive taxonomy, we added communication interventions used specifically in vaccination campaigns to the existing routine vaccination taxonomy. We extracted vaccination campaign communication intervention descriptions from three sources: a targeted literature search of vaccination campaign descriptions; primary observation of vaccination communication in three LMICs (Cameroon, Mozambique and Nigeria); and consultation with LMIC vaccination stakeholders and experts.

### Data source 1: targeted literature search

We built the original routine vaccination taxonomy using intervention descriptions derived from literature review of trials, Medline-indexed literature and grey literature that focused on routine vaccination only [5]. To expand the taxonomy, we conducted a targeted literature search for descriptions of vaccination campaign communication. One author (JK) screened all articles and extracted data. The aim of this literature search was to capture the maximum breadth of communication interventions used in campaigns. We therefore extracted data on all unique examples of vaccination communication interventions until we achieved saturation.

#### Inclusion criteria

We included documents describing any interventions to communicate about vaccines delivered to children in the context of vaccination campaign activities, including descriptions of what could be done in future campaigns (e.g. planning materials). We included descriptions of campaigns related to influenza vaccination (including H1N1) as long as children were specifically included in the targeted population, because these are among the only campaigns conducted in high-income countries (HICs) and our goal was to ensure that this taxonomy was as globally comprehensive as possible. We did not include human papillomavirus vaccine (HPV) (delivered to adolescents).

#### Search strategy

We found that the richest descriptions of campaign communication interventions were policy or programme documents, so we searched for grey literature in key online databases (e.g. PATH Vaccine Resource Library, The Communication Initiative Network). We also drew on a concurrent WHO grey literature review examining a wide range of public health risk communication strategies [20]. Details of our literature search are available in an additional file (see Additional file 1).

We used snowballing to find additional materials cited in references lists of relevant documents and requested references from project partners and vaccination experts from the COMMVAC advisory group.

#### Data extraction

From the included literature, we extracted information about any communication interventions utilised in vaccination campaigns, including:

- description of the intervention;
- content of the communication;
- vaccine/s administered and type of campaign (e.g. measles SIA);
- location of the campaign and country income level; and
- source of the article (e.g. agency website search).

#### Additional searching to establish saturation

To verify whether our search was sufficient, we developed and ran an additional search using Medline (see Additional file 2 for the complete Medline search strategy) and screened results by title and abstract. Of studies identified as potentially relevant, we assessed the full text of a random sample (this sample is outlined in Additional file 1). We found that approximately two thirds of the sampled articles that appeared relevant actually included little or no description of specific communication elements used in the campaigns. From the sampled articles that did include communication details, no unique interventions were identified that had not been seen in the grey literature. Therefore, we determined the taxonomy had reached saturation and we did not continue data extraction from Medline beyond our sample.

### Data source 2: primary field work observation

Three authors (HA, AM, AO) undertook field work in Cameroon (Central and North West Regions), Mozambique (Nampula Province) and Nigeria (Bauchi State in Northern Nigeria and Cross River State in Southern Nigeria). These three countries were selected to provide a varied sample of vaccination communication practices across countries with three different primary languages (French, English, Portuguese) and disease circumstances (e.g. polio status). COMMVAC project resources and research networks were also accessible in these locations, which facilitated the organisation and conduct of this complex field work.

While we were primarily interested in communication interventions used in vaccination campaigns, the field researchers observed both routine and campaign vaccination practices, recording details of communication strategies using a standard form. Details of these studies are described elsewhere [21, 22].

### Data source 3: consultation with stakeholders

The field researchers gathered additional information on communication strategies through discussions with vaccination stakeholders (e.g. funders, planners, implementers, governmental and non-governmental representatives, parents and community members). Interviews, focus groups and analysis of policy documents were used to identify communication strategies in use but which had not necessarily been observed during fieldwork.

The researchers also presented the COMMVAC routine vaccination taxonomy to stakeholders in the LMIC study countries, and at an international workshop organised by COMMVAC (Paris, September 2015) which included senior representatives from LMIC ministries of health and multinational organisations (WHO, Gavi). Stakeholders provided feedback on the taxonomy’s structure and usability as a tool for thinking about communication programmes.

### Categorising the interventions

We compiled the interventions derived from all three data sources into a single database, comprising over 340 interventions, to facilitate categorisation.

We began by categorising the campaign communication interventions according to the routine vaccination taxonomy categories where possible. We then used constant comparison between the routine vaccination taxonomy and the new data to expand, redefine and change categories to more appropriately capture all the communication interventions from both routine and campaign contexts. This process involved iterative discussions within the COMMVAC team and with external stakeholders at the aforementioned COMMVAC workshop.

## Results

Below we report briefly on the data gathered from each source, and then present the comprehensive COMMMVAC taxonomy of communication interventions for childhood vaccination (Table 1).

**Table 1:** The comprehensive COMMVAC taxonomy of communication interventions for childhood vaccination

Purpose	Intervention types
Inform or Educate	Interpersonal communication
Interventions to enable people to understand the meaning and relevance of vaccination to their health and the health of their family or community. Interventions may be tailored to particular populations and can also serve to address misinformation.	e.g. face to face interactions, one-on-one or in groups
	Printed material
	e.g. pamphlets, brochures, fact sheets, media kits
	Mail
	e.g. postcards, letters, newsletters or email
	Phone
	e.g. telephone calls, hotlines or SMS
	Objects, devices or tools
	e.g. printed mugs, t-shirts, magnets or calendars
	Web-based
	e.g. online forums, social media, websites
	School curriculum kits
	e.g. lesson plans, activity booklets, or other materials designed for use in schools
	Community event
	e.g. rallies, vaccination carnivals, health week events
	Edutainment performance
	e.g. song, skit, docudrama or performance on TV, radio, film, theatre
	Mass media advertising
	e.g. notifications or advertisements delivered by newspaper, radio, TV, town criers
	Celebrity spokespeople
	e.g. messages delivered by recognisable or influential people
Remind or Recall	Interpersonal communication
Interventions to remind consumers of required vaccinations and to recall those who are overdue.	e.g. face to face interactions, one-on-one or in groups
	Mail
	e.g. postcards, letters, newsletters or email
	Phone
	e.g. telephone calls, hotlines or SMS
	Objects, devices or tools
	e.g. vaccination cards, printed mugs, t-shirts, magnets or calendars
	Electronic or physical prompts for providers
	e.g. reminders targeting healthcare providers during consultations
Enhance Community Ownership	Community input
Interventions to increase community participation and promote interaction between the community and health services. Interventions may build trust among consumers and generate awareness and understanding of vaccination. Interventions of this nature embrace community involvement in planning, programme delivery, research, social mobilisation, advocacy or governance.	e.g. seeking input or feedback related to intervention design, planning or research
	Community involvement in vaccination programme delivery
	e.g. engagement of members of the community as peer educators, mothers’ support networks, social mobilisers
	Engagement of local opinion leaders
	e.g. faith leaders, local government officials, respected members of a community
	Community coalition
	e.g. community health or ward development committees
	Partnership building
	e.g. vaccine organisers forming partnerships with local businesses, religious centres, community organisations
Teach Skills	Communication training
Interventions focusing on the acquisition of skills related to accessing vaccination services and communicating about vaccination. Such interventions aim to teach parents early parenting skills such as how to find, access and utilise vaccination services. They also include interventions to train parents, communities and health care providers on how to communicate or provide vaccination-related education to others.	e.g. training in communication or education provision skills for community members, volunteers, health professionals, lay health workers or others
	Parenting skills programs
	e.g. early parenting skills training including how to find, access and utilise vaccination services
Provide Support	Interpersonal communication
Interventions, often tailored or personalised, to assist people in addressing specific challenges to vaccination that arise within their day-to-day lives (e.g. social issues such as disagreement within a family regarding vaccinating or emotional issues such as parental anxiety about vaccination). In contrast to interventions to inform or educate, interventions to provide support are more focused on addressing specific challenges faced by parents when making vaccination decisions.	e.g. face to face interactions, one-on-one or in groups
	Phone
	e.g. telephone calls, hotlines or SMS
	Web-based
	e.g. online forums, social media, websites
Facilitate Decision-Making	Decision aids
Interventions that extend beyond informing or educating by presenting all options related to vaccination decision-making in an unbiased and impartial manner. These interventions should explain the decision to be made, provide detailed, evidence-based information about the risks and benefits of vaccination and should help people consider their personal values and options related to the decision to vaccinate their child.	e.g. written or interactive decision aid tools presenting all options and aspects of vaccination decisions
	Decision coaching
	e.g. face to face interactions, one on one or in groups, that guide participants to consider all options, personal values and aspects of vaccination decisions
Enable Communication	Interpreters
Interventions that explicitly and purposefully aim to bridge a communication gap/make communication possible with particular people or groups. This may include translation beyond routine practice in a particular setting, such as translation into local or minority languages, adaptation of materials for a low- or no-literacy population, translation into Braille, or the use of interpreters.	e.g. purposeful engagement of people who speak or sign specific languages
	Translation beyond routine practice
	e.g. translation into local languages, adaptation of materials for a low- or no-literacy population, translation into Braille

From the targeted literature search, we screened over 2000 grey literature documents and 1874 titles and abstracts retrieved from Medline. We extracted descriptions of 283 interventions from 43 documents before we determined that we were no longer identifying unique interventions and had reached saturation (see Additional file 1). From LMIC field work observations and in-country stakeholder consultations we added 58 campaign communication descriptions to the intervention database. Additional stakeholder consultations at the workshop did not add new interventions, but informed the organisational structure and category definitions of the taxonomy.

### The comprehensive taxonomy of communication for childhood vaccination

Like the routine vaccination taxonomy, the comprehensive COMMVAC taxonomy (Table 1) organises the range of vaccination communication interventions according to the *primary purpose* of the communication. The taxonomy’s seven communication purpose categories are: ‘Inform or Educate’; ‘Remind or Recall’; ‘Enhance Community Ownership’; ‘Teach Skills’; ‘Provide Support’; ‘Facilitate Decision Making’ and ‘Enable Communication’. These purposes remained unchanged from the routine vaccination taxonomy because they fully captured all the newly identified campaign interventions. However, in response to stakeholder feedback, we re-ordered these purposes to move the ‘Enhance Community Ownership’ category higher in the list (from appearing last in the routine vaccination taxonomy) so the most relevant and frequently-used intervention categories appear first. Additionally, some stakeholders found it difficult to understand which interventions would be included in the less common purpose categories, or felt there was too much overlap between these categories and the ‘Inform or Educate’ category. We addressed this issue by revising the definitions of the categories ‘Teach Skills’, ‘Provide Support’, ‘Facilitate Decision Making’ and ‘Enable Communication’.

Within each purpose category, the routine vaccination taxonomy grouped the communication into *intervention types,* or the modes or routes through which each communication purpose is enacted or implemented. The intervention types in the comprehensive taxonomy now accommodate the addition of campaign communication interventions through updated terminology and the addition of new intervention types. For example, the wording of some intervention types now reflects commonly-used terminology (e.g. “audiovisual/performance” was changed to “edutainment performance”, a term LMIC stakeholders recognised) and clarifies the categories for multi-lingual stakeholders. The comprehensive taxonomy also includes new intervention types not identified in routine vaccination contexts, such as school curriculum kits for lessons involving vaccination information and community-based reminders for upcoming campaigns.

Table 1 presents the comprehensive COMMVAC taxonomy with seven communication intervention purposes and their definitions, intervention types for each purpose, and examples of each intervention type, drawn from routine communication and campaign communication.

**Fig. 2:**
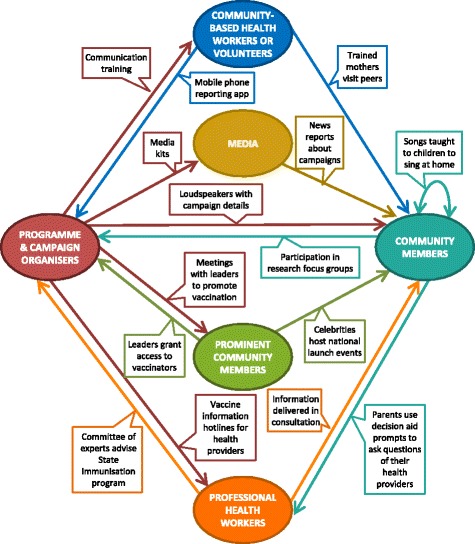
The multi-directional nature of vaccination communication: examples of the actors and channels involved

The comprehensive taxonomy reflects the multi-directional nature of communication [23]. In the routine vaccination taxonomy, we delineated three target groups for communication: parents, communities and health providers. However, we found that in campaign communication, the actors and channels or directions of communication were more diverse. The routine vaccination taxonomy targets were too restrictive and they unintentionally implied that the communication was unidirectional. We therefore removed the targets from the comprehensive taxonomy to allow for the fluidity of campaign communication. As a supplemental exploration of the varied actors and channels involved in campaigns, we developed a visual map to illustrate a small selection of these complex interactions (Fig. 2).

## Discussion

Communication is often complex. A single intervention may include printed material to inform or educate as well as a face-to-face session intended to teach skills, or a postcard may include vaccine information as well as a reminder about an upcoming campaign. These examples could be appropriately categorised into more than one purpose or intervention type. The aim of the taxonomy is not to create wholly exclusive categories, but to help conceptualise communication interventions as a range of potential solutions to address needs or problems. This is why the entry point for the taxonomy is through the communication’s intended purpose. It is also important to acknowledge that communication strategies do not need to address all purposes at all times, and the taxonomy is not itself a “menu” of options that are all of equal effectiveness and appropriateness. For many of the interventions included in the taxonomy, there is limited or inconclusive evidence of their effectiveness. More primary studies and systematic reviews of frequently utilised interventions are needed.

Below, we will discuss our observations about the differences and similarities between communication in campaigns and communication for routine vaccination, as well as the ways in which the taxonomy may be utilised, as identified by the international experts consulted at the Paris workshop.

### Observations about campaign and routine vaccination communication

Campaigns take a more multi-faceted approach to communication design and delivery than routine vaccination communication. In routine vaccination communication, the primary actors are parents, communities and health providers. However, as the examples in Fig. 2 suggest, communication in campaigns involves a range of actors. The organisational structure, scale of the communication and individuals in each role may vary depending on the setting, but most campaigns describe communication taking place between many of these actors in a multi-directional way. Mapping a communication network such as the one illustrated in Fig. 2 may help people in different roles identify the range of channels available to send out or solicit communication.

Campaigns may use a particularly broad range of communication channels and actors because communication and social mobilisation efforts tend to be allocated more resources in campaigns than in routine vaccination activities [21, 22]. The intensity of communication strategies may also be a response to the clear focus of campaigns to obtain ambitious results in short periods in large populations, particularly in the case of campaign responses to disease outbreaks (e.g. campaigns to address yellow fever outbreaks [19]). But while they may face resource limitations, routine vaccination communication programmes can apply lessons and concepts from campaigns on a smaller scale, such as encouraging community involvement and promoting two-way input and feedback.

In the interventions we identified, communication involving community engagement tended to be more common in campaigns than in routine vaccination. Such interventions were also more frequently discussed in LMIC settings than in HICs. Given that peer-to-peer communication and other health initiatives aimed at communities can influence social norms and behaviour [24, 25], such interventions may be a valuable approach for routine vaccination in both LMICs and HICs to address issues like vaccine hesitancy or pockets of resistance [8, 15, 26, 27].

Communication strategies in the ‘Inform or Educate’ category were the most frequently used and diverse in format and medium, in both campaigns and routine vaccination. This is not surprising, since the most basic definition of communication is information delivery between parties. However, the range of unique and inventive ‘Inform or Educate’ strategies employed by campaigns is noteworthy. One new intervention type we added to this category was school curriculum kits [28]. These were multi-media packages of vaccination materials, designed for implementation by teachers in schools. We were not previously aware of interventions of this nature in the context of routine childhood vaccination. This may be because children themselves are not frequently the target of communication about routine vaccinations, whereas in campaigns, children are targeted so they can spread awareness and information about campaign activities to their families and communities [29–31]. The urgency of a campaign encourages a broad approach to reaching and engaging people throughout society.

‘Remind or Recall’ interventions were used frequently in both contexts, though community-wide reminders were almost exclusively observed in campaigns due to the personal nature and individual schedule of routine vaccination. Communication training interventions in the ‘Teach Skills’ category also appeared in routine vaccination and campaigns, with a wider variety of people receiving the training in campaigns (e.g. priests trained to deliver vaccination messages at mass) [29].

Communication strategies to ‘Provide Support’ or ‘Facilitate Decision-Making’ were very rarely or never observed or recorded in campaign literature, and except for a rare instance of face-to-face decision coaching [32, 33], are also largely absent from routine vaccination communication in LMICs. This may be because these interventions require more time to implement, or because they are often individually tailored. The typically large scale and short timeframes of campaigns most likely preclude resource allocation to more specialised interventions.

### How can the taxonomy help with communication implementation?

The taxonomy is an organisational tool that can be used in a number of ways. First, it provides a standardised way to think and speak about vaccination communication. Second, researchers and funders can use the taxonomy to see the range of communication interventions that are being used in practice, synthesise the evidence available for these interventions and identify important evidence gaps.

Finally, as we established in our consultations with LMIC stakeholders, the taxonomy can be utilised by programme planners or people who make decisions about which communication interventions to implement. By presenting communication in terms of purpose, the taxonomy encourages people to view communication options through a problem-solving lens, mapping context-specific barriers to intervention purposes. For example, if misinformation is an issue, producing myth-busting interventions to ‘Inform or Educate’ may be important as might building trust through interventions to ‘Enhance Community Ownership’. The taxonomy also allows planners to consider potential alternative options that serve the same purpose, but may be cheaper or require less skilled staff. Additionally, the taxonomy may help planners determine whether there are other communication purposes that should be addressed in their setting.

### Strengths and limitations

This taxonomy is, to our knowledge, the first framework to organise the full range of communication interventions for childhood vaccination. It brings together communication used in routine vaccination efforts as well as campaigns and includes interventions from high-, middle- and low-income countries. The taxonomy reflects the multi-directionality of communication and the range of actors and channels involved. Qualitative data collection was undertaken in only three countries, which were all in Africa. This is a potential limitation as we may have observed additional interventions in other global regions. However, selecting three countries from the same region facilitated cross-country comparisons in other aspects of the COMMVAC project, which would have been more difficult if the settings were in vastly different global regions. Furthermore, our targeted literature search was global in scope in order to ensure the taxonomy’s comprehensiveness and relevance to different settings.

Early versions of the taxonomy were tested for clarity and usability with researchers and programme planners in LMICs. They found it conceptually complex and occasionally challenging to translate into other languages, which informed some of our subsequent changes. They appreciated the taxonomy’s focus on the intended purpose of potential communication options, which helps link interventions to the underlying communication problem. While it is not a self-contained menu of options, the taxonomy can play an important role in planning and decision-making regarding vaccination communication interventions when taken together with other sources of information, such as systematic reviews and information about the acceptability, feasibility and resource requirements of different interventions for a particular context [34–37].

A limitation is that the taxonomy presents a range of possible strategies but does not include evidence of their effects. Systematically reviewing the evidence for each intervention was outside the scope of this project. Furthermore, high-quality evidence on the full range of interventions does not yet exist [38, 39]. The taxonomy helps to identify interventions that are in use but may be untested – and so may be used by researchers and funders to map evidence gaps and focus future efforts on establishing whether these strategies are effective.

## Conclusion

The COMMVAC 2 comprehensive taxonomy of communication interventions for childhood vaccination is a unique framework providing the first overarching view of the scope of childhood vaccination communication interventions, presenting them according to their primary purpose. The taxonomy can be used to expand the range of potential strategies considered and implemented by programme planners or map and prioritise research efforts.

## Additional files

Additional file 1: Literature search details (Data Source 1). Diagrams and descriptions of literature search results. (DOCX 40 kb)

Additional file 2: Medline search strategy. Medline literature search strategy. (DOCX 16 kb)

## Abbreviations

COMMVAC: Communicate to vaccinate
HIC: High-income country
HPV: Human papillomavirus
LMIC: Low- or middle-income country
SIA: Supplementary immunisation activities
