# Supplementary material for: The comprehensive ‘Communicate to Vaccinate’ taxonomy of communication interventions for childhood vaccination in routine and campaign contexts
Source: BMC Public Health. 2017 May 10;17:423. doi: 10.1186/s12889-017-4320-x (PMC5424416; doi:10.1186/s12889-017-4320-x)
Supplement: Supplementary file 1 — Literature search details (Data Source 1). Diagrams and descriptions of literature search results. (DOCX 40 kb) [file 12889_2017_4320_MOESM1_ESM.docx]

## Additional File 1 - Literature search details (Data Source 1)

**Grey literature and snowballing search results**

46 included documents

USAID IMMUNIZATIONbasics database (<http://www.immunizationbasics.jsi.com/>): We used the site's Resources index and screened all publications listed under “Communication.”

PATH Vaccine Resource Library (<http://www.path.org/vaccineresources/topics.php>): We browsed this database using its "Explore Topics" index. We screened all articles categorised under “Vaccines and Immunization”, “For advocates and policymakers”, and “Emerging and epidemic diseases”. We also searched using the phrases “Mass vaccination campaign communication” and “vaccination communication”.

The Communication Initiative Network (<http://www.comminit.com/global/>): We searched the website using the phrase "mass vaccination campaign communication".

WHO review: We screened the review's included documents for materials specifically focusing on infectious disease outbreaks, pandemic influenza, and public health disasters/emergencies more generally.

**Medline
(July 12, 2014)**

Non-duplicate results retrieved (n=1874)

Excluded from abstract and title (n=1516)

**Relevant and included articles (n=13)**

Articles excluded from full text (n=32)

*SAMPLE*: Articles sought in full text (n=45)

n=358

**Medline search and sampling results**
