# Supplementary material for: The comprehensive ‘Communicate to Vaccinate’ taxonomy of communication interventions for childhood vaccination in routine and campaign contexts
Source: BMC Public Health. 2017 May 10;17:423. doi: 10.1186/s12889-017-4320-x (PMC5424416; doi:10.1186/s12889-017-4320-x)
Supplement: Supplementary file 2 — Medline search strategy. Medline literature search strategy. (DOCX 16 kb) [file 12889_2017_4320_MOESM2_ESM.docx]

## Additional File 2 – Medline search strategy

Strategy - Ovid MEDLINE(R) In-Process & Other Non-Indexed Citations, Ovid MEDLINE(R) Daily, Ovid MEDLINE(R) and Ovid OLDMEDLINE(R) 1946 to Present – Searched 12th July 2014

1. Immunization/

2. Immunization Schedule/

3. Immunization, Secondary/

4. Immunotherapy/

5. exp Immunotherapy, Active/

6. Immunization Programs/

7. exp Vaccines/

8. (vaccin$ or revaccinat$ or immuniz$ or immunis$ or immunother$ or inoculat$ or innoculat* or prophyla*).tw.

9. or/1-8

10. (mass or campaign* or eradic*).ti,ab.

11. 9 and 10

12. ("periodic intensification of routine immuni?ation" or "supplementary immuni?ation activities" or "national immuni?ation" or NIDS).ti,ab.

13. Mass Vaccination/

14. 11 or 12 or 13

15. exp Child/

16. exp Infant/

17. Child Care/

18. Infant Care/

19. exp Perinatal Care/

20. exp Parents/

21. (child$ or infant$ or newborn? or neonat$ or preschool* or primary school* or baby or babies or toddler$ or parent$ or mother$ or father$).tw.

22. or/15-21

23. exp Communication/

24. ((health or patient* or mediated or facilitated or augmentative or alternative or total or simultaneous or manual or mass or face-to-face or one-to-one or one-on-one or oral or cultural or risk or intervention* or interaction* or program* or skill* or aid* or tool* or board* or device* or system* or barrier*) adj1 communication).mp.

25. (communicat* or messag* or verbal* or nonverbal* or written or writing or reading or language or speech or speak* or spoken or talk* or conversation* or voice or visual-perception or feedback or listen* or negotiat* or notify* or notification or remind* or narrat* or music* or humor or humour or humorous or adverti* or persua* or interpreting or interpreters or interpret*-service or translat* service* or translating).hw,ti.

26. (readability or intelligibility or credibility).mp.

27. (disclos* or trust* or truth* or deceiv* or deception or misinform*).hw,ti.

28. exp Interpersonal Relations/

29. hospital patient relations/

30. community institutional relations/

31. ((professional or physician or doctor or clinician or nurse or provider) adj1 (patient or client or family)).tw.

32. ((health or patient or client) adj (education or knowledge or promotion)).mp.

33. exp health promotion/

34. ((education* or teaching or learning or instruction* or training or skills or online or web* or internet or video* or multimedia or multi-media) adj1 (intervention* or session* or course* or program* or material* or package* or module* or demonstration or method* or process*)).mp.

35. (self adj (teaching or education or instruction)).mp.

36. ((media adj3 campaign*) or (promotion adj1 program*) or (community based adj3 intervention*) or (awareness adj3 (rais* or increas*))).tw.

37. marketing.mp.

38. ((family or office or work* or school or faith or church) adj based).tw.

39. (educational status or literacy).mp.

40. ((improv* or increas* or enhanc* or patient) adj3 (understanding or comprehension)).tw.

41. (information* adj (service* or center* or centre* or system* or dissemination or seeking or retrieval or transfer* or campaign* or provision or aid or material* or sheet* or pack*)).mp.

42. ((patient or client or health or medical or drug or written or print* or visual* or provid* or present*) adj2 inform*).mp.

43. (((inform* or message* or communicat* or effect* or gain or positive or negative) adj2 fram*) or ((verbal or oral or written or text or data or numerical or statistical or visual or graphic* or pictorial or audio* or video* or multimedia or multi-media or narrative) adj (format* or presentation* or display*))).mp.

44. (counsel* or ((social or carer* or caregiver* or care giver* or patient*) adj1 support*) or psychosocial or ((social or pastoral or spiritual) adj care) or religio* or chaplaincy or behavior modification or behaviour modification).mp.

45. (counsel*ing session* or ((support or peer or self-help or self-care) adj2 (intervention* or group* or program*))).mp.

46. ((social or community) adj2 network*).mp.

47. (self-care or self-management).mp.

48. (motivat* or incentive* or goal*).mp.

49. exp Communications Media/

50. ((mass or communication* or electronic or digital or multi or print* or social or new) adj media).tw.

51. ((print* adj (material* or based)) or paper-based or written material* or (paper adj1 pen*) or publication* or newsletter* or brochure* or booklet* or pamphlet* or leaflet* or flyer* or handout* or poster* or illustrat* or picture* or pictogram*).mp.

52. (radio or television or audiovisual or video* or tape recording* or cassette* or cd-rom* or dvd* or motion picture* or movie* or cinema* or multimedia or hypermedia or telephon* or phone or phones or sms or short message* or text message* or i-pod* or ipod* or i-pad* or ipad* or mp3 player* or hotline* or answering service* or internet or web* or online or on-line or blog* or telemedicine or telehealth or telecare or (virtual adj (reality or world or environment*))).mp.

53. ((electronic or e-) adj (mail or prescri* or health or game*)).mp.

54. exp computer systems/

55. software/

56. (computer* adj1 (system* or network* or program* or terminal* or interfac* or interact* or handheld or intervention* or therapy or graphic* or simulation* or searching or mediated or based or tailored or communication or assisted instruction)).mp.

57. (touch screen or digital assistant* or pda or blackberry or mobile-device* or laptop* or notebook computer* or computer* or netbook*).mp.

58. (((automat* or interactive*) adj3 (telephon* or phone or phones or voice or hotline* or hot line*)) or ((voice or speech) adj (response or recognition or messag* or system* or technolog*))).mp.

59. (cultural* adj (competen* or sensitiv* or appropriate)).mp.

60. ((cultural* or linguistic* or language) adj3 (service* or care or intervention* or message*)).mp.

61. (participation or advocacy or consumer* or empower*).mp.

62. exp decision making/

63. (decision adj (making or support or aid*)).mp.

64. exp informed consent/

65. (informed adj (consent or choice* or decision*)).tw.

66. ((patient or person or family or client) adj (cent*red or focus*ed or oriented)).mp.

67. (therapeutic adj (relation* or alliance*)).mp.

68. or/23-67

69. Developing Countries.sh,kf.

70. Africa/ or Asia/ or Caribbean/ or West Indies/ or South America/ or Latin America/ or Central America/

71. (Africa or Asia or Caribbean or West Indies or South America or Latin America or Central America).tw.

72. (Afghanistan or Albania or Algeria or Angola or Argentina or Armenia or Armenian or Azerbaijan or Bangladesh or Benin or Byelarus or Byelorussian or Belarus or Belorussian or Belorussia or Belize or Bhutan or Bolivia or Bosnia or Herzegovina or Hercegovina or Botswana or Brazil or Bulgaria or Burkina Faso or Burkina Fasso or Upper Volta or Burundi or Urundi or Cambodia or Khmer Republic or Kampuchea or Cameroon or Cameroons or Cameron or Camerons or Cape Verde or Central African Republic or Chad or China or Colombia or Comoros or Comoro Islands or Comores or Mayotte or Congo or Zaire or Costa Rica or Cote d'Ivoire or Ivory Coast or Cuba or Djibouti or French Somaliland or Dominica or Dominican Republic or East Timor or East Timur or Timor Leste or Ecuador or Egypt or United Arab Republic or El Salvador or Eritrea or Ethiopia or Fiji or Gabon or Gabonese Republic or Gambia or Gaza or Georgia Republic or Georgian Republic or Ghana or Grenada or Guatemala or Guinea or Guiana or Guyana or Haiti or Honduras or India or Maldives or Indonesia or Iran or Iraq or Jamaica or Jordan or Kazakhstan or Kazakh or Kenya or Kiribati or Korea or Kosovo or Kyrgyzstan or Kirghizia or Kyrgyz Republic or Kirghiz or Kirgizstan or Lao PDR or Laos or Lebanon or Lesotho or Basutoland or Liberia or Libya or Macedonia or Madagascar or Malagasy Republic or Malaysia or Malaya or Malay or Sabah or Sarawak or Malawi or Mali or Marshall Islands or Mauritania or Mauritius or Agalega Islands or Mexico or Micronesia or Middle East or Moldova or Moldovia or Moldovian or Mongolia or Montenegro or Morocco or Ifni or Mozambique or Myanmar or Myanma or Burma or Namibia or Nepal or Netherlands Antilles or Nicaragua or Niger or Nigeria or Muscat or Pakistan or Palau or Palestine or Panama or Paraguay or Peru or Philippines or Philipines or Phillipines or Phillippines or Papua New Guinea or Romania or Rumania or Roumania or Rwanda or Ruanda or Saint Lucia or St Lucia or Saint Vincent or St Vincent or Grenadines or Samoa or Samoan Islands or Navigator Island or Navigator Islands or Sao Tome or Senegal or Serbia or Montenegro or Seychelles or Sierra Leone or Sri Lanka or Solomon Islands or Somalia or Sudan or Suriname or Surinam or Swaziland or South Africa or Syria or Tajikistan or Tadzhikistan or Tadjikistan or Tadzhik or Tanzania or Thailand or Togo or Togolese Republic or Tonga or Tunisia or Turkey or Turkmenistan or Turkmen or Uganda or Ukraine or Uzbekistan or Uzbek or Vanuatu or New Hebrides or Venezuela or Vietnam or Viet Nam or West Bank or Yemen or Zambia or Zimbabwe).tw,sh.

73. ((developing or less* developed or under developed or underdeveloped or middle income or low* income or underserved or under served or deprived or poor*) adj (countr* or nation? or population? or world or state*)).ti,ab.

74. ((developing or less* developed or under developed or underdeveloped or middle income or low* income) adj (economy or economies)).ti,ab.

75. (low* adj (gdp or gnp or gross domestic or gross national)).tw.

76. (low adj3 middle adj3 countr*).tw.

77. (lmic or lmics or third world or lami countr*).tw.

78. transitional countr*.tw.

79. or/69-78

80. 14 and 22 and 68 and 79

81. 14 and 22 and 68

82. limit 81 to yr="1990 -Current"
